# Therapy-induced senescent cancer cells as bidirectional regulators of antitumor immunity and resistance in the tumor microenvironment

**DOI:** 10.1038/s41419-026-08688-z

**Published:** 2026-04-01

**Authors:** Minji Choi, Daeun Lee, Wen-Hao Yang, Jong-Ho Cha

**Affiliations:** 1 Department of Biomedical Sciences, College of Medicine, Inha University, Incheon, Republic of Korea; 2 Program in Biomedical Science and Engineering, Graduate school, Inha University, Incheon, Republic of Korea; 3 Graduate Institute of Biomedical Sciences, China Medical University, Taichung, Taiwan, ROC; 4 Biohybrid Systems Research Center, Inha University, Incheon, Republic of Korea

**Keywords:** Cancer microenvironment, Tumour immunology

## Abstract

Cancer immunotherapy has markedly improved patient outcomes, particularly when combined with conventional treatments such as chemotherapy, radiotherapy, and targeted therapy. Following these therapies, however, a subset of cancer cells can enter a senescent state, ceasing proliferation while remaining metabolically active and persistent within tissues. Such therapy-induced senescent cancer cells (TISCCs) significantly influence antitumor immune responses. TISCCs can enhance tumor immunogenicity by presenting neoantigens and activating innate immune pathways. Conversely, they can also promote T-cell immune evasion and therapeutic resistance, ultimately leading to an immunosuppressive tumor microenvironment. This dual role of TISCCs represents a critical determinant of immunotherapy efficacy, making their precise modulation a major challenge for optimizing combination treatment strategies. In this review, we comprehensively examine the opposing roles of TISCCs in antitumor immunity and highlight emerging therapeutic approaches that mitigate TISCC-driven immune suppression and improve the overall efficacy of immunotherapy-based combination regimens.

## Facts

- Senescent cancer cells exhibit dual immunological phenotypes depending on their temporal and contextual state, initially promoting antitumor immunity but eventually contributing to immune evasion and tumor progression.
- Therapy-induced senescence generates distinct immune-modulatory programs through secretory factors (SASP), checkpoint ligands, and extracellular vesicles, reshaping the tumor microenvironment in both stimulatory and suppressive directions.
- The transition from immune-stimulatory to immune-suppressive TISCC states remains poorly understood, with key molecular switches and regulatory pathways (e.g., SASP dynamics, IFN-JAK-STAT, checkpoint evolution) yet to be fully delineated.
- The therapeutic potential of targeting TISCCs is underexplored, including strategies such as immunogenic senescence priming, SASP modulation (senomorphics), checkpoint blockade during the immunosuppressive phase, and selective elimination via senolytics.
- Parallels between TISCCs and dormant disseminated tumor cells suggest a convergence in immune evasion strategies and recurrence mechanisms, warranting investigation into shared regulatory circuits and combined therapeutic vulnerabilities.

## Introduction

In the era of immuno-oncology, cancer treatment has evolved from merely eradicating malignant cells to reprogramming the complex ecosystem that exists between cancer cells and the immune system [1–3]. Although immune checkpoint inhibitors (ICIs) have greatly contributed to cancer treatment, only a subset of patients achieve durable responses, while many patients experience limited or transient benefits [4–6]. Emerging evidence suggests that therapeutic outcomes are determined not only by immune activation itself, but also by how preceding cytotoxic therapies such as chemotherapy, radiotherapy, and targeted therapy reshape the tumor microenvironment (TME) and influence subsequent immune dynamics [7–9].

These cytotoxic modalities, though intended to eliminate cancer cells, often leave behind a residual population of TISCCs [10, 11]. These cells are metabolically active yet non-proliferative, persisting long after treatment has ceased [12]. Initially regarded as benign remnants of therapy, TISCCs are now recognized as key modulators of immune activity [13]. By releasing a diverse array of inflammatory and immunoregulatory factors collectively known as the senescence-associated secretory phenotype (SASP), they paradoxically act as both amplifiers of immune activation and orchestrators of immune evasion [14–16]. On one hand, TISCCs enhance tumor immunogenicity by increasing antigen presentation and activating innate immune pathways, thereby converting immunologically “cold” tumors into “hot,” immune-reactive states [17–19]. On the other hand, persistent SASP signaling, together with the upregulation of inhibitory immune checkpoints such as programmed death-ligand 1 (PD-L1) and PD-L2, promotes T-cell exhaustion and establishes a chronically inflamed yet immunosuppressed TME [20–22].

This duality positions TISCCs at a critical intersection between tumor suppression and immune escape [22, 23]. Despite their growing biological and therapeutic significance, the senescence–immunity axis remains underexplored within current clinical frameworks, which continue to emphasize direct cytotoxicity over immune modulation [24–26]. Deciphering the molecular mechanisms that determine whether TISCCs act as a facilitator or suppressor of antitumor immunity will be essential for the rational design of next-generation combination strategies that integrate senescence biology into immunotherapy [27–29].

In this review, we discuss the emerging concept of TISCCs as bidirectional regulators of tumor immunity. We discuss their immunostimulatory and immunosuppressive mechanisms, their implications for therapeutic resistance and recurrence, and potential strategies to either harness or eliminate senescent cancer cells to improve the efficacy and durability of immunotherapy.

## Therapy-induced senescence in cancer

**Fig. 1:**
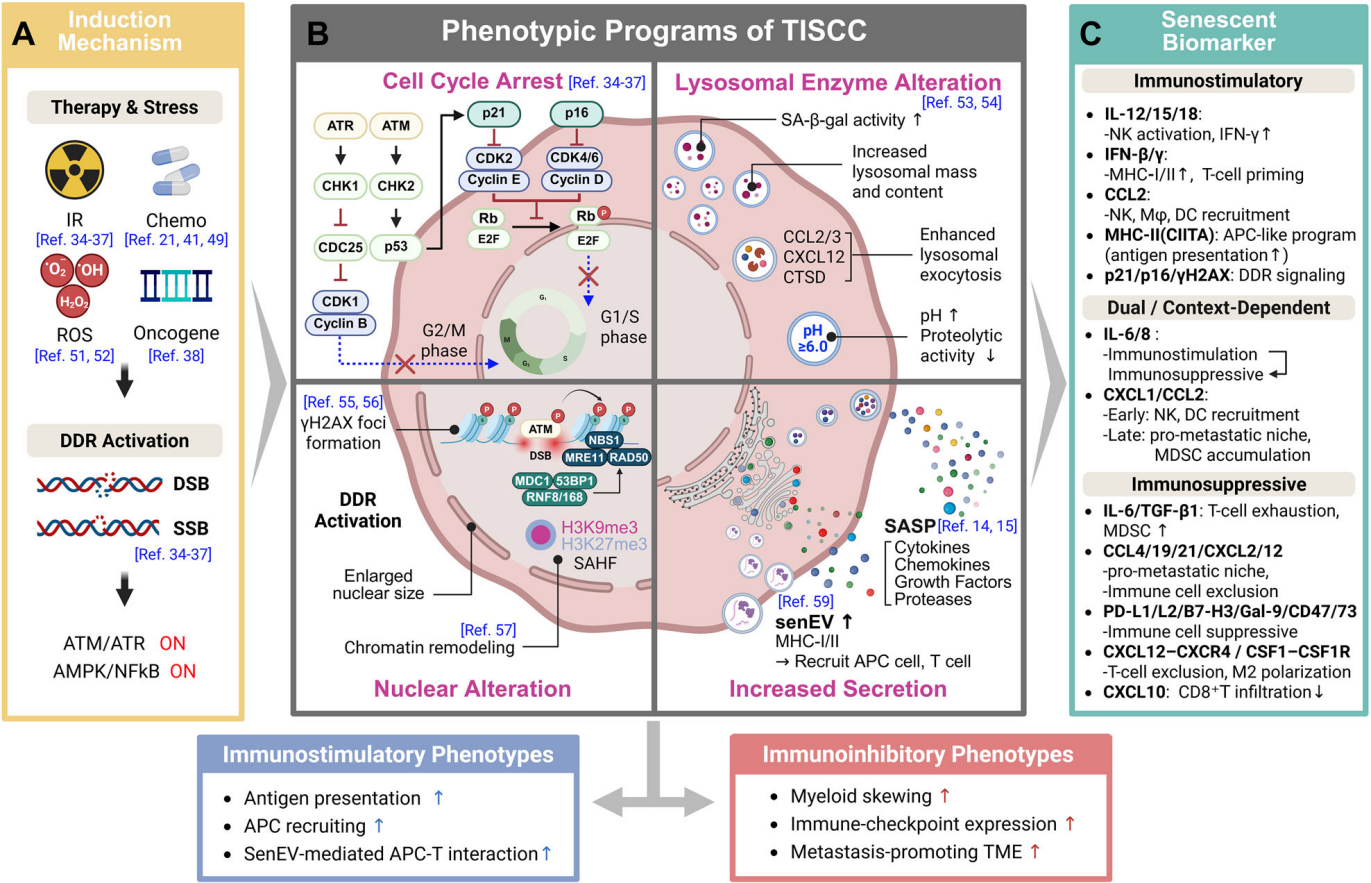
Induction, phenotypic characterization, and immunomodulatory features of TISCCs. **A** Stress conditions such as chemotherapy, irradiation, or oncogene activation induce a persistent DDR that activates the ATM and ATR kinases. These kinases subsequently engage AMPK and the NF-κB pathway, collectively initiating the cellular senescence program. **B** TISCCs display hallmark phenotypic programs characterized by cell-cycle arrest via the p53–p21 and p16–RB axes, lysosomal and nuclear remodeling, and increased secretion of SASP factors, including cytokines, chemokines, and senEVs, which convey immunogenic ligands and MHC molecules. **C** Senescent biomarkers are categorized as immunostimulatory, dual or context-dependent modulators, and immunosuppressive factors. Together, these panels illustrate the complex immune landscape sculpted by TIS and the sequential formation and functional polarization of TISCCs. This illustration was created using BioRender.com.

Cell senescence is an irreversible state of terminal cell-cycle arrest that is mechanistically and phenotypically distinct from programmed cell death. In both normal and malignant cells, senescence can be induced by various intracellular and extracellular stressors; representative inducers include DNA damage, reactive oxygen species (ROS), and abnormal oncogenic signaling pathways [30]. Particularly, conventional anticancer therapies like radiation therapy and chemotherapy can induce potent genotoxic and oxidative stress responses, leading tumor cells to undergo senescence as part of the treatment response [13, 26, 31]. Growing evidence links this treatment-induced senescence (TIS) to treatment response, disease recurrence, and the reconfiguration of the tumor immune microenvironment, establishing TISCC as a central axis in clinical oncology that influences treatment design and sequencing [29, 32] (Fig. 1).

[24, 26]

### Mechanisms of TISCC induction

TISCC most commonly arises from activation of the DNA damage response (DDR) [33]. Ionizing radiation (IR) generates double-strand breaks (DSBs) in tumor DNA, activating the damage sensors ataxia-telangiectasia mutated (ATM) and ataxia telangiectasia and Rad3-related (ATR) [34, 35]. These signals propagate through checkpoint kinase 1/checkpoint kinase 2 (CHK1/CHK2) to engage tumor-suppressor axes principally p53–p21 and p16–retinoblastoma protein (RB) to enforce durable G1/S and/or G2/M arrest, thereby committing cells to a senescent fate [36, 37]. In parallel, stress-responsive nodes including adenosine 5′-monophosphate (AMP)-activated protein kinase (AMPK), mechanistic target of rapamycin (mTOR), mitogen-activated protein kinase p38 (p38 MAPK), and nuclear factor kappa B (NF-κB) reprogram metabolism and transcription, thereby stabilizing the senescent phenotype and licensing expression of the SASP [38–41]

The propensity to induce senescence is modality- and context-dependent. The DNA-damaging agent doxorubicin elicits a robust senescence program in p53-wild-type cells, whereas p53-deficient counterparts fail to mount an equivalent response and remain senescence-associated beta-galactosidase (SA-β-gal)-negative, underscoring p53 as a central regulator of TIS initiation and maintenance [42]. Microtubule-targeting agents such as paclitaxel can likewise induce senescence through prolonged mitotic arrest and subsequent mitotic slippage, which triggers DNA damage and activates the p53–p21 pathway [43–46]. Additionally, paclitaxel-induced chromosome missegregation also leads to the formation of micronuclei, whose rupture releases genomic DNA into the cytosol and activates the cGAS-STING pathway [47, 48]. These events are reflected by increased SA-β-gal activity and upregulation of inflammatory SASP mediators [e.g., interleukin-1α (IL-1α), IL-6, matrix metallopeptidase-3 (MMP-3), MMP9, C-X-C motif chemokine ligand 1 (CXCL1)] [49].

Beyond genotoxic stress, accumulation of ROS serves as a potent senescence trigger [50, 51]. In cancer stem cells (CSCs), hydrogen peroxide (H₂O₂)-induced oxidative stress activates the p53/p21 pathway to drive senescence, concomitantly reducing tumor-initiating capacity and revealing a therapeutic opportunity to exploit redox vulnerabilities [52].

These pathways simultaneously trigger enforced activation of cell-cycle checkpoints, sustained DDR signaling, and stress-responsive transcriptional programs, which are the most reliable indicators of senescence. On this basis, a multi-parameter biomarker panel can effectively define the state of TISCCs and establish the foundation for function-centered analysis.

### Biomarkers of TISCCs

SA-β-gal is one of the most widely used surrogate markers of cellular senescence and can be readily detected in cultured cells and tissue samples. Mechanistically, its increased activity in senescent cancer cells is largely attributed to an expansion and remodeling of the lysosomal compartment, characterized by increased lysosomal mass and hydrolase content, and often accompanied by altered luminal pH. These changes are thought to reshape overall lysosomal proteolytic capacity, a feature commonly associated with the senescent phenotype [53, 54]. In fixed specimens, p16 (CDKN2A/INK4a) and p21 (CDKN1A/CIP1/WAF1) serve as canonical indicators of cell-cycle arrest, while DDR foci (phosphorylated histone H2AX (γH2AX) and tumor suppressor p53-binding protein 1 (53BP1)) report the persistent DNA-damage signaling characteristic of senescent states [55, 56]. In some contexts, this state is accompanied by chromatin remodeling, including lamin B1 depletion and formation of senescence-associated heterochromatin foci (SAHF). Taken together, these markers enable reliable identification of cellular senescence [57].

Complementing these state markers is an analysis of the secretome/vesiculome. TISCCs elaborate a characteristic SASP, releasing inflammatory cytokines [e.g., IL-1α, IL-6, IL-8], chemokines [e.g., CXCL1, CXCL10, C-C motif chemokine ligand 2 (CCL2), CCL8], growth factors, and proteases [e.g., MMP3, MMP9] [49, 58], while concurrently shedding senescence-associated extracellular vesicles (senEVs). Together, these secretory and vesicular cues propagate inflammatory signaling and calibrate immune-cell recruitment and function, exerting system-level effects on the TME and reflecting the effector dimension of the senescent phenotype [59, 60].

Importantly, marker constellations and their temporal dynamics are functionally informative. DDR/type I IFN-skewed profiles tend to associate with upregulated antigen-processing/presentation machinery and increased antigen-presenting cell (APC) trafficking, whereas NF-κB-dominant, SASP-high states correlate with myeloid skewing and immune-checkpoint ligand induction. Building on this marker–function mapping, the next section systematically examines the immune-regulatory programs driven by TISCCs.

## Immunostimulatory roles of TISCCs

**Fig. 2:**
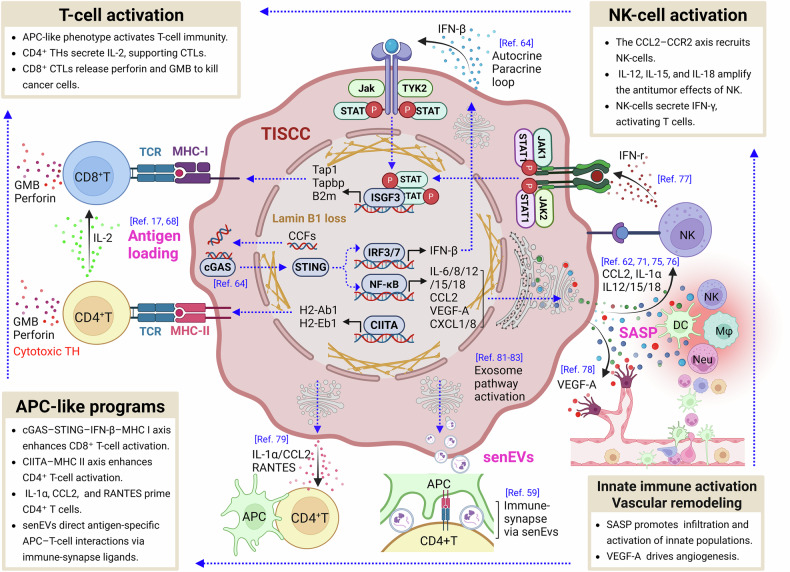
TISCCs are an immune-activating phenotype that reshapes the TME toward immunostimulation. TISCCs remodel the tumor microenvironment into an immune-permissive niche and coordinate the activation of both innate and adaptive immunity. Activation of the cGAS–STING–NF-κB pathway induces VEGF-A, promoting angiogenesis that facilitates immune-cell infiltration, whereas SASP factors recruit NK cells, macrophages, and DCs. NK-cell activity is amplified through the CCL2–CCR2 axis and IL-12, IL-15, and IL-18 signaling, resulting in IFN-γ release, which bridges innate and adaptive responses. Together with senEVs, TISCCs enhance T-cell immunity by providing immune-synapse ligands that strengthen antigen-specific APC–T-cell interactions. The IFN–JAK–STAT axis upregulates MHC-I to prime CD8⁺ T cells, while the CIITA–MHC-II axis, supported by IL-1α, CCL2, and RANTES, drives CD4⁺ T-cell activation. Abbreviations: Neu, neutrophil; GMB, granzyme B; Mφ, macrophage. This illustration was created using BioRender.com.

Current cancer immunotherapy has moved beyond merely boosting T-cell activity to remodeling the TME so that tumors become intrinsically susceptible to immune attack. Central to this shift is the strategy of converting immunologically “cold” tumors into “hot” tumors, which has heightened interest in cellular states that share features with immunogenic cell death (ICD). Within this context, TISCCs have emerged as enablers of antitumor immunity. Through the SASP, TISCCs release inflammatory cytokines and chemokines that recruit and activate innate immune effectors within the tumor. Beyond recruitment, TISCCs can amplify multiple stages of the immune response by enhancing antigen processing and presentation, reprogramming the TME toward an immune-permissive niche, and delivering precise, extracellular vesicle (EV)–mediated cues that coordinate dendritic cell (DC)–T-cell crosstalk and strengthen effector priming. This section delineates these immunostimulatory programs and examines how TISCCs can be leveraged as therapeutic assets, particularly within ICI-based combination strategies and rational treatment sequencing (Fig. 2).

### Augmented antigen presentation by TISCCs via enhanced immunogenicity

Immune surveillance of senescent cells is mediated by multiple effector populations, including natural killer (NK) cells, macrophages, and T cells and operates most effectively when targets are readily visible to the immune system [61, 62]. For T-cell-mediated recognition in particular, such visibility requires competent antigen processing and presentation via major histocompatibility complex (MHC) class I and II pathways [63]. TISCCs meet these prerequisites: they upregulate MHC-I and MHC-II, thereby improving detection by CD8⁺ and CD4⁺ T cells, respectively.

Mechanistically, loss of lamin B1 destabilizes the nuclear envelope and permits leakage of cytosolic chromatin fragments (CCFs), which activate cGAS–STING signaling [64]. Upon activation, STING recruits TBK1–IKK complexes and activates both NF-κB and IRF3/7 pathways, leading to induction of pro-inflammatory SASP factors and transcription of IFN-β [41, 65]. The released IFN-β acts in both autocrine and paracrine manners to remodel the tumor microenvironment [64], establishing an interferon-responsive milieu that extends beyond senescent tumor cells. Through IFNAR signaling, IFN-β activates the JAK–STAT pathway and forms the ISGF3 complex, driving transcription of interferon-stimulated genes [66]. This IFN-driven remodeling enhances tumor-associated antigen availability, facilitates their capture by antigen-presenting cells (APC), and ultimately promotes cytotoxic CD8⁺ T-cell activation and tumor recognition [67].

Proteomic data support this trajectory: in plasma-membrane–enriched fractions from senescent cells, Marin et al. reported increased MHC-I–linked antigen-processing/presentation components; concordantly, type I IFN activates antigen-processing pathways and elevates expression of their gene products. An activated MHC-I system enables presentation of self-derived and neoantigenic peptides at the tumor cell surface, promoting CD8⁺ T-cell priming and effector activation, a foundation of immune-mediated tumor control [17]. Beyond MHC-I enhancement, TISCCs can acquire myeloid APC-like transcriptional programs. In therapy-induced senescent lymphoma, key myeloid transcription factors (Purine-rich nucleic acid binding protein 1 (PU.1), CCAAT/enhancer-binding protein-β (C/EBPβ), Activator Protein-1 (AP-1)) are engaged, inducing the class II transactivator (CIITA) and strongly upregulating MHC-II genes [e.g., H2-Ab1, H2-Eb1] [68]. Functionally, this confers the capacity to present antigen to CD4⁺ T cells, broadening and sustaining antitumor responses.

Taken together, TISCCs are not inert, growth-arrested remnants of therapy. By augmenting MHC-I/II–mediated antigen presentation, they actively participate in and can amplify CD8⁺ and CD4⁺ T-cell surveillance. These features position TISCCs as conditional co-stimulators of antitumor immunity whose immunogenicity can be therapeutically leveraged. Strategies that tune their antigen-presenting capacity and integrate this with ICI-based regimens provide a rational path to enhance immune-mediated tumor control.

### Immune reprogramming of the tumor microenvironment by TISCCs

TISCCs can function as active immunoregulators that reshape the TME. Central to this activity is the SASP—a repertoire of immunostimulatory mediators, including IL-6, IL-8, TNF-α, CCL2, CXCL1, CXCL8, and type I interferons—that promotes the recruitment and activation of innate immune populations such as NK cells, monocytes, macrophages, and neutrophils [62, 69, 70]. Among these cues, CCL2–CCR2 signaling efficiently attracts NK cells and macrophages [71, 72], whereas CXCL1/CXCL8–CXCR2 engagement increases neutrophil infiltration [73]. Notably, TISCC-derived CCL2 can recruit NK cells without any change in natural killer group 2, member D (NKG2D)-ligand expression on tumor cells; once recruited, NK cells recognize pre-existing ligands and mediate tumor cell killing, establishing an effective immune axis [74]. In parallel, IL-12 [75], IL-15 [76], and IL-18 [62] released from TISCCs potentiate NK-cell cytotoxicity and IFN-γ production, thereby amplifying antitumor effects [77].

Concurrently, TISCCs exhibit marked hypersensitivity to exogenous immune stimuli, particularly IFN-γ [62]. Upregulation of IFNGR1 and activation of the downstream STAT1-MHC class I pathway increase transcription of genes governing antigen processing and presentation, creating a hypersensitized IFN-γ–responsive state that triggers robust antigen-presentation programs even in response to minute amounts of IFN-γ from T and NK cells in the TME. Consistent with this, IFNGR-deficient TISCCs show impaired immune clearance despite intact SASP output, indicating that responsiveness to extrinsic cytokine signals rather than SASP alone is a decisive determinant of immune surveillance in senescent tumor cells [62].

Beyond cytokine responsiveness, TISCCs can help overcome structural barriers in the TME. Ruscetti et al. reported that TISCCs secrete angiogenic factors, including vascular endothelial growth factor A (VEGF-A), which increase intra-tumoral vascular density and facilitate CD8⁺ T-cell infiltration [78]. This vascular remodeling underpins the conversion of immune-evasive “cold” tumors into immune-responsive “hot” tumors and creates conditions favorable for improved responses to ICIs.

Emerging evidence further suggests that TISCCs can initiate CD4⁺ T-cell–mediated immunity. Oncogene-induced senescent hepatoma cells secrete CCL2, IL-1α, and regulated upon activation, normal T-cell expressed and secreted (RANTES), thereby recruiting DCs and activating antigen-specific CD4⁺ T cells; the resulting Th1-oriented IFN-γ response contributes to tumor suppression [79]. In addition, senescent fibroblasts are known to upregulate human leukocyte antigen class II (HLA-II) and can present antigens to elicit cytotoxic CD4⁺ T cells that directly eliminate senescent targets via granzyme B and perforin [80]. By analogy, and given the shared features of TIS, it is plausible that a similar HLA-II–restricted CD4⁺ cytotoxic pathway operates in TISCCs. These findings extend the traditional CD8-centric model and highlight the functional plasticity with which TISCCs engage multiple immune axes.

In summary, TISCCs exert potent immunostimulatory effects through innate-cell recruitment, heightened cytokine sensitivity, vascular remodeling that enables lymphocyte access, and induction of CD4⁺ and CD8⁺ T-cell responses. Collectively, these properties argue that TISCCs are not mere byproducts of therapy but strategic targets capable of reprogramming the tumor immune landscape and amplifying antitumor immunity.

### TISCC-derived extracellular vesicles as active immune modulators

In their crosstalk with the extracellular milieu, senescent cells influence immunity not only through soluble SASP factors but also via extracellular vesicles (EVs) [81–83]. The SASP Atlas, which catalogs proteins secreted by senescent cells across diverse triggers and lineages, underscores that SASP composition and function vary markedly with cellular origin and inducing stress [83].

TISCCs release senescence cell–derived EVs (senEVs) that are not metabolic waste but active immunoregulatory agents. Functionally, senEVs have been shown to be both necessary and sufficient to drive immune-mediated tumor control. Ziglari et al. reported that TISCC-derived EVs promote intratumoral APC recruitment and activation, thereby amplifying tumor-specific immune responses [59]. In this study, senEV cargo includes ligands and signaling molecules that support immune-synapse formation between DCs and CD4⁺ T cells, facilitating precise, antigen-specific T-cell activation. Importantly, when EV secretion is selectively inhibited while soluble SASP is preserved, tumors exhibit markedly reduced infiltration of MHC class II⁺ APCs, a shift toward ineffective, neutrophil-dominant inflammation, and dampened CD4⁺ and CD8⁺ T-cell responses. These observations argue that senEVs are indispensable effectors of TISCC immunogenicity and the key contributors to tumor control.

Collectively, TISCCs are active immunomodulators that can initiate and coordinate antitumor immunity through complementary outputs: (i) enhanced antigen presentation [62, 84], (ii) trafficking of innate immune cells [79, 85], (iii) remodeling of the TME toward an immune-permissive state [64], and (iv) senEV-mediated, antigen-specific instruction of APC–T-cell interactions [86]. Whereas soluble SASP broadly recruits and activates immune cells within tumors, senEVs provide the precision layer that orchestrates APC–T-cell crosstalk. Together, these facets position TISCCs not as passive targets but as therapeutically addressable sources of “danger signals” that can be leveraged to enhance immune surveillance and antitumor efficacy.

## Immunosuppressive features of TISCCs

**Fig. 3:**
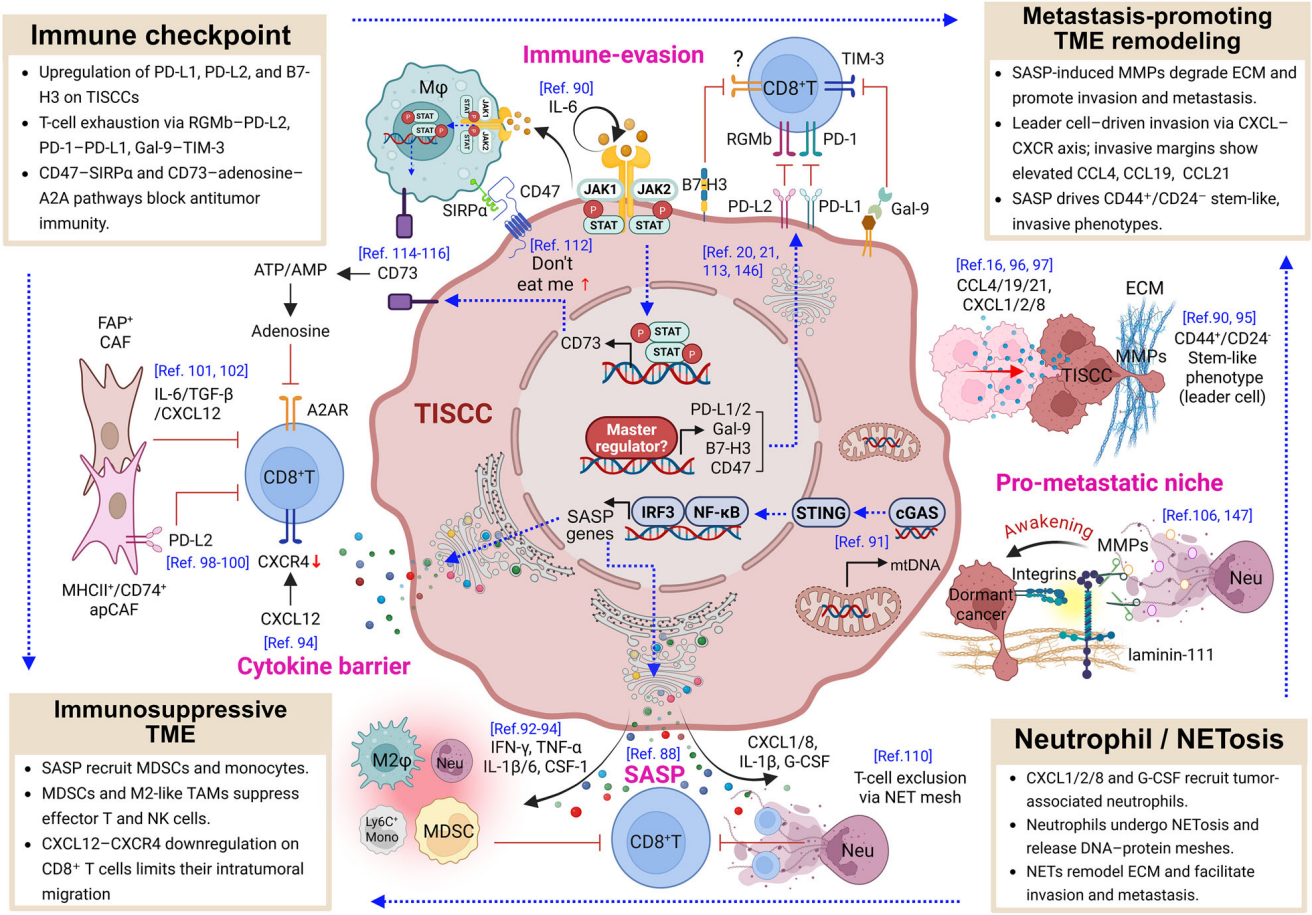
TISCCs have an immune-evasive and metastasis-permissive phenotype that reshapes the TME toward immunosuppression. As TISCCs persist, they progressively reprogram the TME toward an immune-suppressive and pro-metastatic state. SASP-derived cytokines, including IL-6, IL-8, CCL12, and CSF1, recruit immunosuppressive myeloid subsets such as MDSCs, Ly6C⁺ monocytes, and M2-like macrophages. These, in conjunction with chemokine gradients and NETs, establish both physical and functional barriers that exclude CD8⁺ T cells from the tumor core. TISCCs also upregulate a repertoire of immune checkpoint ligands (PD-L1, PD-L2, B7-H3, CD47), activating adaptive and innate immune evasion programs. CD73-mediated adenosine production and PD-L2–RGMb interactions further inhibit T-cell activation and promote T-cell exhaustion. Simultaneously, SASP cytokines and MMPs remodel the extracellular matrix, induce EMT, and foster a stem-like tumor phenotype conducive to invasion and dissemination. TISCC-driven NETs may facilitate pre-metastatic niche formation and awaken dormant tumor cells and/or senescent cells, contributing to recurrence. Altogether, these mechanisms generate an immune-excluded, metastasis-favorable ecosystem. This illustration was created using BioRender.com.

Acute inflammation can facilitate tumor recognition and prime antitumor immunity, whereas chronic inflammation accelerates malignancy and disrupts immune control. In this respect, TISCCs may be immunostimulatory early after treatment but can transition to an immunosuppressive role when they persist in the TME. Immediately after therapy, SASP cues recruit and activate innate immune effectors to promote tumor clearance [62, 87]. Over time, however, a sustained SASP skews the TME toward chronic inflammation and immune dysfunction, ultimately diminishing the responsiveness to immunotherapy. Pro-inflammatory cytokines such as IL-6, IL-8, and TNF-α sustain unresolved inflammatory responses [88], reshaping the immune landscape toward myeloid remodeling, immune-checkpoint ligand induction, and T-cell exhaustion, thereby impairing antitumor immunity [89]. In parallel, senescent tumor cells can engage IL-6/IL-8 autocrine loops that increase invasiveness and metastatic potential, mechanistically linking immune disruption to tumor progression [90]. On this basis, the section systematically delineates the TISCC-mediated programs that establish and sustain an immunosuppressive TME (Fig. 3).

### Immunosuppressive reprogramming of the TME by TISCCs

TISCCs can chronically remodel the TME into an immunosuppressive state, thereby weakening antitumor immunity and reducing therapeutic responsiveness. In a total-body irradiation (TBI) mouse model, senescence induction led to decreased infiltration and impaired function of CD8⁺ T cells, accompanied by the accumulation of myeloid-derived suppressor cells (MDSCs) and Ly6C^+^ monocytes [28]. Notably, mitochondrial DNA (mtDNA) released from senescent tumor cells activated the cGAS–STING–NF-κB signaling pathway, which enhanced the suppressive activity of MDSCs and further reinforced the immunosuppressive TME [91].

Transcriptomic profiling revealed that monocytes within the senescent milieu exhibited upregulation of immunosuppressive gene signatures, including *Thbs1, Vegfa, Tgm2, Cd44* [28], consistent with reduced anti-PD-L1 responsiveness. Single-cell RNA sequencing further demonstrated that myeloid populations adopted more pronounced suppressive phenotypes. Furthermore, treatment with the senolytic ABT263 increased intratumoral CD8⁺ T-cell numbers and decreased suppressive gene expression in myeloid cells, implicating TISCCs as central contributors to the maintenance of immune evasion and underscoring their role in sustaining a chronically immunosuppressive TME [78].

Beyond general myeloid activation, TISCCs suppress antitumor immunity by SASP-driven macrophage polarization toward M2-like phenotypes and by establishing chemokine barriers that restrict T-cell entry. In hepatocellular carcinoma, senescent cancer cells secrete a SASP enriched in IFN-γ, TNF-α, IL-6, and IL-1β [92]. Unlike non-senescent counterparts, which favor M1-like (CD80⁺, CD86⁺) programs, the senescent SASP skews differentiation toward M2-like (CD163⁺, CD206⁺) macrophages, thereby reinforcing tumor-promoting myeloid circuits [93]. Mechanistically, sustained CXCL12 signaling reduces CXCR4 expression on CD8⁺ T cells, thereby limiting their intratumoral migration. Similarly, in colorectal cancer, senescent cells produce CXCL12 and colony-stimulating factor 1 (CSF1) to establish a “cytokine barrier” that protects non-senescent tumor cells from CD8⁺ T-cell infiltration [94]. Mechanistically, CXCL12–CXCR4 signaling redirects T-cell trafficking and limits entry into tumor nests, while CSF1 drives M2 polarization of monocytes, jointly suppressing T-cell activation. Importantly, pharmacologic or genetic neutralization of CXCL12 and CSF1 has been shown to restore T-cell infiltration and reduce tumor burden, identifying both signaling axes as promising therapeutic targets [94].

In summary, TISCCs orchestrate immune evasion through multiple hierarchical mechanisms: activation of suppressive myeloid cell populations, reprogramming of tumor-supporting macrophages, and chemokine-mediated T-cell exclusion. Targeting these inhibitory circuits offers a rational strategy to overcome immunotherapy resistance in tumors enriched with senescent cells.

### Formation of a metastasis-promoting TME by TISCCs

Senescent cancer cells have traditionally been regarded as tumor-suppressive due to their irreversible cell-cycle arrest. However, accumulating evidence now shows that, despite their proliferative arrest, these cells can actively remodel the TME in ways that promote invasion and metastasis. Central to this pro-metastatic reprogramming is the SASP, which not only remodels the extracellular matrix (ECM) but also reprograms immune cell functions, thereby enhancing cancer cell motility and metastatic potential.

The SASP can also induce stem-like programs in a context-dependent manner. In breast cancer, autocrine loops involving IL-6 and IL-8 drive acquisition of a CD44⁺/CD24⁻-like stem-cell phenotype, increasing motility and invasiveness and thereby facilitating metastasis [90]. Remarkably, in papillary thyroid carcinoma (PTC), senescent cancer cells act as leader cells at the invasive front, coordinating the collective invasion of neighboring non-senescent cancer cells [95]. This process is driven by the CXCL–CXCR signaling axis, particularly CXCL12–CXCR4 interactions, and is characterized by elevated expression of CCL4, CCL19, CCL21, and CXCL2 at the invasive margins. Similarly, in prostate cancer, SASP components such as MMPs, IL-6, CXCL1, and CXCL8 contribute to the establishment of a pro-metastatic niche by promoting ECM degradation and modulating immune cell infiltration [16, 96, 97].

Senescence within the tumor stroma further conditions the TME for metastasis. Following chemotherapy in pancreatic ductal adenocarcinoma (PDAC), senescent cancer-associated fibroblasts (senescent CAFs) are central drivers of immunosuppressive remodeling. A subset termed antigen-presenting CAFs (apCAFs) is defined by MHC-II/CD74 expression [98]. These cells display low levels of CD80/CD86 [99], which limits professional antigen-presenting cell function, and they serve as a major source of PD-L2 [100]. In parallel, fibroblast activation protein-α (FAP)⁺ CAFs-derived CXCL12, IL-6, and TGF-β1 exclude intratumoral CD8⁺ T cells and drive T-cell exhaustion [101, 102]. Collectively, these changes enhance resistance to ICIs and generate a malignant TME in which immunosuppression and metastatic progression are tightly linked.

Together, senescent cancer cells and stromal components cooperate to construct an invasion- and metastasis-permissive TME through SASP-mediated ECM remodeling, activation of chemokine networks, induction of stem-like phenotypes, and immune suppression. These insights highlight senolytic interventions and SASP modulation as promising strategies to suppress metastasis and restore responsiveness to immunotherapy.

### Potential association between TISCCs and neutrophils/NETosis

TISCCs secrete a wide range of immunomodulators through SASP [103]. Among these, prominent SASP components such as CXCL8 (IL-8), CXCL1, G-CSF, IL-1β, and IL-6 are potent recruiters and activators of neutrophils, suggesting that TISCCs may serve as central organizers of intratumoral neutrophil infiltration [95, 104, 105].

A useful parallel can be drawn from studies on dormant cancer cells. These growth-arrested cells can be reactivated by neutrophil-driven NETosis, thereby promoting metastatic outgrowth and recurrence [106, 107]. Neutrophil extracellular traps (NETs) are fibrous lattices composed of DNA–histone complexes and proteins such as neutrophil elastase (NE) and MMP-9; they can remodel the ECM and deliver motility and proliferative cues to tumor cells [108, 109]. By analogy, TISCCs may similarly respond to NET-derived cues, increasing their potential to re-enter the cell cycle or facilitate relapse and distant metastasis following therapy.

A key concept emerging from these findings is the functional synergy between SASP and NETosis. SASP factors [such as IL-8, CXCL1, and G-CSF] recruit and prime neutrophils, while NETosis, in turn, suppresses antitumor immunity by obstructing CD8⁺ T-cell infiltration and disrupting immune-synapse formation through the physical NET meshwork and associated proteolytic enzymes [110]. The result can be a self-reinforcing inhibitory loop in which SASP biases neutrophils toward NETosis and NETs, in turn, suppress T-cell–mediated responses, thereby strengthening tumor immune evasion and reducing responsiveness to ICIs.

Although a direct causal link between TISCCs and NETosis has yet to be established, mechanistic parallels from dormant-cell models, together with the strong neutrophil-attracting capacity of TISCCs, support the plausible existence of a senescent cancer–neutrophil–NETosis axis. To test this hypothesis, several lines of investigation would be informative: (i) Spatial co-localization of NET markers [citrullinated histone H3 (Cit-H3), myeloperoxidase (MPO)–DNA] with SASP factors in TISCC-rich tumors. (ii) Quantitative assays of NET formation, ECM cleavage, and T-cell suppression in TISCC–neutrophil co-culture systems. (iii) Intervention studies using peptidylarginine deiminase 4 (PAD4) inhibitors, DNase, and CXCR1/2 antagonists to determine whether NET inhibition restores T-cell infiltration and antitumor efficacy.

In summary, it is conceivable that TISCC-derived SASP induces NETosis and that NETs reciprocally reinforce immunosuppression and tumor reactivation. Therapeutic strategies combining SASP modulation with NET inhibition may thus offer a promising avenue to prevent post-treatment recurrence and improve the efficacy of ICI-based immunotherapies.

### Immune checkpoint activation and immune-evasion programs in TISCCs

Cellular senescence represents a terminal stress-response program that permanently halts the proliferation of damaged cells while coupling them to immune surveillance and clearance mechanisms mediated through both innate and adaptive immunity [70, 111]. In tumors, however, senescent cancer cells often deviate from this physiological pathway. When they persist following therapy, these cells rewire their interactions with the immune system to favor survival, with aberrant upregulation of immune-checkpoint molecules serving as a key mechanism.

CD47, which is markedly upregulated on senescent tumor cells [112]. CD47 engages Signal-regulatory protein α (SIRPα) on macrophages to deliver a potent “do not-eat-me” signal through activation of Src homology 2 domain-containing phosphatase 1 (SHP-1), thereby blocking cytoskeletal remodeling and inhibiting phagocytosis. Moreover, N-terminal pyroglutamate modification of CD47 by glutaminyl-peptide cyclotransferase (QPCT) and QPCT-like (QPCTL) enhances its affinity for SIRPα, further consolidating the phagocytic blockade. This pathway enables long-term persistence of senescent tumor cells and reinforces the formation of an immunosuppressive TME [112].

In parallel, T-cell immune checkpoints are broadly activated in senescent tumor cells. These cells exhibit strong upregulation of PD-L1, PD-L2, and B7 homolog 3 (B7-H3), all of which contribute to adaptive immune evasion. The endoplasmic reticulum–associated protein dolichyl-diphosphooligosaccharide–protein glycosyltransferase subunit 1 (RPN1) plays a critical role by mediating PD-L1 glycosylation, which enhances its stability and surface expression. Consequently, PD-L1 more effectively engages programmed cell death protein 1 (PD-1) on CD8⁺ T cells, reinforcing immune escape [20]. During drug-induced senescence, PD-L2 is likewise upregulated through NF-κB signaling, and PD-L2⁺ senescent cells secrete CXCL1, CXCL2, and IL-6 to recruit myeloid-derived suppressor cells (MDSCs), accelerating immunosuppressive remodeling. Notably, PD-L2 interacts not only with PD-1 but also with repulsive guidance molecule b (RGMb) on T cells, creating a PD-1–independent inhibitory axis that further restricts T-cell activation and cytotoxicity [21].

Secreted checkpoint mediators also play a critical role in systemic immune suppression. In a temozolomide-induced senescence model of B16-F10 melanoma, expression and secretion of galectin-9 were markedly elevated, suppressing the activity of T cells, NK cells, and macrophages while expanding regulatory T cells and M2 macrophages. Acting as a soluble checkpoint molecule, galectin-9 propagates systemic immune suppression beyond the local TME, thereby impairing the clearance of senescent tumor cells [113].

Another crucial immunosuppressive mechanism involves the CD73–adenosine axis. In the senescent tumor microenvironment, IL-6 secreted by senescent cancer cells activates the JAK–STAT3 pathway in both tumor and immune cells, leading to transcriptional upregulation of CD73. Elevated CD73 expression enhances the enzymatic conversion of extracellular ATP/AMP into adenosine, which in turn suppresses T-cell activation through the A2A receptor. Importantly, pharmacologic blockade of CD73 can restore CD8⁺ T-cell-mediated antitumor immunity, identifying this pathway as a reversible target for therapeutic intervention [114–116]

Collectively, TISCCs function as potent hubs of immunosuppression within the TME by simultaneously activating multiple checkpoint pathways. Although they no longer proliferate, these cells effectively evade immune surveillance, providing a transient protective niche that enables residual proliferative tumor cells to escape immune control and regenerate. Consequently, TISCCs may serve as a major cellular source of tumor recurrence following therapy. The coordinated induction of diverse checkpoint mechanisms suggests the existence of an upstream master regulator orchestrating this immune-evasion network. Identifying and targeting this regulator should be prioritized in efforts to prevent recurrence and restore sensitivity to ICIs.

## Time-aware therapeutic strategies targeting TISCCs

Despite the robust antitumor efficacy demonstrated by senolytic and senomorphic interventions in preclinical models [28, 117, 118], their clinical translation has been largely disappointing—often producing inconsistent or suboptimal outcomes (Table 1). We propose that this translational gap arises from a critical yet overlooked factor: the temporal dynamics of TISCCs, which current trial designs fail to adequately capture.

**Table 1:** Representative clinical trials of senotherapies (senomorphics/senolytics) and immunotherapy combinations.

NCT ID (Alias)	Condition	Intervention	Phase/Design		Toxicity assessment
NCT04037462	NSCLC	Dexamethason + Pembrolizumab (PD-1)		Phase 1b/2	Yes—safety monitored as trial criterion (“unacceptable toxicity” discontinuation), but results not posted yet.
NCT05724329	Resectable HNSCC (Neoadjuvant ± Adjuvant)	Dasatinib + Quercetin + Tislelizumab (PD-1)		Phase 2	Yes—trial explicitly evaluates “efficacy and safety”, but no posted results.
NCT06355037	Metastatic TNBC (Chemoresistant)	Dasatinib + Quercetin + Chemotherapy		Phase 2	Yes—trial explicitly evaluates “efficacy and safety”, but no posted results.
NCT05358639	HGSC & TNBC	Olaparib + Navitoclax (BCL-2/XL inhibitor)		Phase 1	Yes—primary goal includes DLT/MTD/RP2D; results pending.
NCT00445198	SCLC & Solid Tumors	Navitoclax (BCL-2/XL inhibitor)		Phase 1/2a	Yes—dose-dependent thrombocytopenia is the major toxicity / DLT signal.
NCT01562873	Breast cancer (incl. TNBC or Inflammatory breast cancer; pSTAT3+ Archival tissue required)	Ruxolitinib (JAK1/2 inhibitor; IL-6/JAK/STAT3 pathway blockade)		Phase 2	Yes—clinical activity limited; trial reports safety monitoring (generally described as tolerable in publications), but objective responses are minimal.
NCT01195922	Resectable HNSCC (Presurgical window/Neoadjuvant setting)	Rapamycin (mTORC1 inhibitor)		Phase 2; presurgical window	Yes —“well tolerated”, no grade 4 or unexpected toxicities reported.

TISCCs are not a static cell population; they function more as a dynamic immunological rheostat. In the immediate aftermath of cytotoxic therapy, they boost antigen presentation and promote immune activation. However, over time, they transition into a chronically immunosuppressive state driven by SASP secretion, fostering tumor immune evasion and progression. This dynamic shift implies that applying a static therapeutic intervention to such a temporally fluid process may be fundamentally flawed. For instance, premature senolysis could eliminate TISCCs before their immune-activating potential is realized, while delayed senomorphic treatment may fail to reverse a deeply entrenched suppressive microenvironment.

**Fig. 4:**
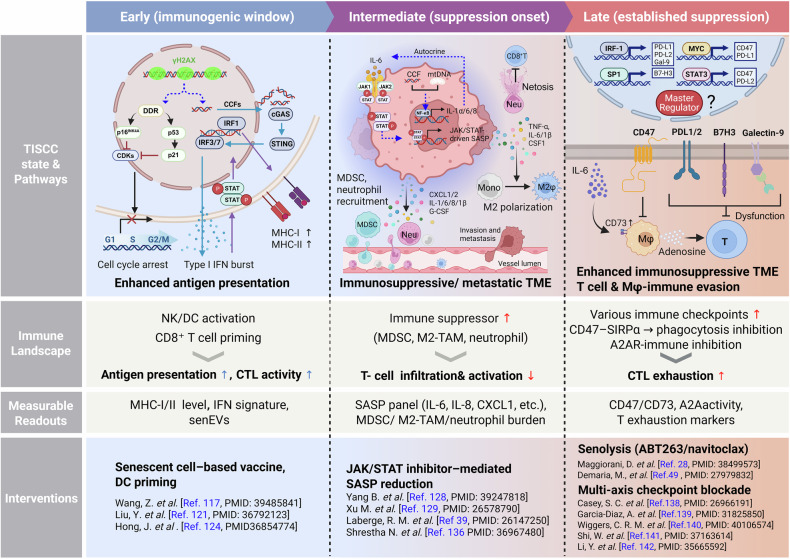
Time-dependent transition of TISCC-associated immune landscapes and therapeutic intervention strategies. Following anticancer therapy, TISCCs initially adopt an immune-activating phenotype that promotes antitumor immunity. Over time, however, these cells transition into an immune-suppressive state that dampens immune surveillance and promotes tumor escape. This temporal trajectory is marked by dynamic changes in measurable parameters, including MHC expression levels, infiltration by MDSCs, M2-polarized macrophages, and neutrophils, as well as upregulation of immune checkpoint ligands. Therapeutic interventions can be strategically aligned with this timeline. Early-phase immunogenic TISCCs can be leveraged to maximize the immunogenic window. This is followed by senomorphic interventions to restrain immunosuppressive SASP output, multi-axis checkpoint blockade to reverse immune evasion, and ultimately, senolytic therapies to eliminate residual TISCCs and prevent sustained immunosuppression or tumor relapse. This illustration was created using BioRender.com.

Addressing this issue requires a paradigm shift—from indiscriminately targeting senescent cells to implementing a Time-Aware Therapeutic Framework. This approach aligns specific interventions with the evolving immunologic profile of TISCCs. By precisely timing the transition from “maximizing immunogenicity” to “removing suppressive burdens,” this sequential strategy offers a novel means to convert the double-edged sword of TISCCs into a decisive therapeutic advantage (Fig. 4).

In the early phase, cytotoxic therapies such as radiation or chemotherapy activate the cGAS–STING and type I interferon pathways in TISCCs, thereby enhancing antigen processing and presentation and stimulating DC activation, which together boost cytotoxic T lymphocyte (CTL) responses [64, 67, 119, 120]. Taking advantage of this transient immunogenic window, senescent cell–based vaccines or DCs primed by senescent cells have been shown in preclinical models to amplify STING-dependent CTL responses and suppress recurrence and metastasis [17, 121–124]. Clinically, a short-term induction of senescence followed by vaccination or ICI therapy represents a rational sequencing approach. Given that the composition of the SASP and the resulting immune responses vary among tumor types and treatments, this “immunogenicity-first” strategy may be particularly effective in poorly inflamed solid tumors [78, 125, 126].

To extend this window and preemptively delay the shift toward suppression, senomorphic modulation can selectively attenuate chronic immunosuppressive SASP signaling while preserving the initial immunostimulatory effects[127]. For example, JAK/STAT inhibitors [e.g., ruxolitinib] and mTOR inhibitors [e.g., rapamycin] both attenuate the expression of immunosuppressive SASP factors [IL-6, IL-8, CXCL1] [39, 117, 128], limiting chronic inflammation [129], secondary senescence propagation [130], T-cell exhaustion [131], and myeloid skewing [132]. In combination with vaccines or ICIs, this approach preserves beneficial priming while dampening inhibitory cues.

As TISCCs persist and begin to orchestrate a more complex inhibitory microenvironment, effective control often requires multi-axis checkpoint blockade. For instance, combining PD-1/PD-L1 inhibition with blockade of the PD-L2–RGMb axis can neutralize PD-1–independent suppression [21]; CD47 blockade (or QPCTL inhibition) restores macrophage phagocytosis [133]; and antagonism of the CD73–A2A adenosine pathway relieves metabolic constraints on T cells [114]. In addition, disialylated ganglioside GD3 has been identified as a senescence-specific immune checkpoint (SIC), opening a route to counter NK evasion [134]. The therapeutic principle is to match the breadth of TISCC-driven inhibitory circuits with commensurately broad but mechanism-guided combinations.

Finally, once TISCCs have accumulated and established a dominant immunosuppressive state, senolytic therapy serves as a critical intervention to eliminate the cellular source of resistance. The BCL-2 inhibitor ABT263 (navitoclax) selectively eliminates senescent cells [31, 135], thereby suppressing the accumulation of MDSCs and Ly6C⁺ monocytes and downregulating immunosuppressive genes such as *Thbs1* and *Vegfa* [28]. This treatment restores intratumoral CD8⁺ T-cell infiltration and function, and when combined with anti–PD-L1 therapy, leads to significant tumor growth delay and improved survival. Additional targeting of Ly6C⁺ monocytes further mitigates senescence-induced immune resistance [28]. Within the tumor stroma, BCL-2–dependent senescent CAFs amplify an IFN-γ-responsive SASP cytokine axis [e.g., IL-6, TGF-β1, and CXCL10] that suppresses CD8⁺ T-cell activation, and their elimination enhances responsiveness to ICIs [101]. Both genetic and pharmacological senolysis have been shown to restore anti–PD-L1 responses impaired by TISCCs accumulation.

Taken together, therapeutic strategies targeting TISCCs should adopt an integrated and time-aware framework built on four key principles. First, maximize the early immunogenic window to fully exploit the transient phase of immune activation following therapy. Second, modulate suppressive SASP signaling using senomorphic agents to preserve beneficial immune stimulation while limiting chronic inflammation. Third, apply multi-axis checkpoint blockade to counteract the diverse inhibitory pathways simultaneously activated by TISCCs. Finally, eliminate residual senescent cells through senolytic approaches to prevent long-term immune suppression and tumor relapse. Although much of the current evidence remains preclinical, the convergence of mechanistic insights and therapeutic outcomes provides a strong rationale for precisely timed, combination-based clinical trials to evaluate these sequential and synergistic treatment strategies [28, 39, 117, 121, 124, 128, 129, 136–142].

## Conclusion and perspective

Combination regimens centered on ICIs have improved clinical outcomes, yet many patients still face immune resistance and relapse. As synthesized in this review, TISCCs sit at the core of both the problem and the opportunity. Far from passive remnants of cytotoxic therapy, TISCCs are context-dependent regulators that can either amplify or suppress antitumor immunity within the TME. Radiation and chemotherapy activate the cGAS–STING–type I interferon axis, heightening antigen processing and presentation [64, 143, 144]. This immunogenic signal is often eclipsed by the parallel upregulation of inhibitory checkpoints and a pro-inflammatory SASP [145]. In TISCCs, broad increases in PD-L1/PD-L2 and B7-H3 are frequently observed [20, 21, 146], and the CD47–SIRPα pathway (potentiated by QPCTL-dependent modification) together with the CD73–A2A adenosine axis consolidate macrophage phagocytosis blockade and T-cell exhaustion [114, 133]. Despite this dynamic biology, clinical eligibility for ICIs still relies largely on pretreatment tumor mutational burden and baseline PD-L1, which can miss checkpoint dynamics that emerge in response to standard therapy and the appearance of TISCCs. Incorporating post-therapy reassessment of checkpoint trajectories into real-world decision-making is therefore warranted.

Beyond immune suppression, TISCCs may also contribute to relapse through reactivation circuits. Dormant cancer cells can be awakened by neutrophil-driven NETosis; NETs remodel extracellular matrix and activate integrin signaling to break dormancy [106, 147]. Senescent tumor cells share phenotypic features with dormancy (SA-β-gal activity, cellular enlargement, proliferation arrest) and can recruit neutrophils via SASP chemokines such as IL-8 and CXCL1, thereby promoting NETosis [70, 108]. Whether this axis directly promotes TISCC re-proliferation remains a key mechanistic question.

While preclinical models have provided a compelling rationale for the anti-tumor potential of senolytics and senomorphics, most clinical trials to date have failed to deliver significant therapeutic benefits (Table 1) [148, 149]. These translational challenges are often attributed to the inherent heterogeneity of TISCCs across different malignancies and the limitations of conventional models in recapitulating the complex human tumor–immune dynamics [150–153]. However, we argue that these failures are not necessarily evidence of biological insufficiency in the senescence-targeting concept itself. Instead, they reflect fundamental shortcomings in clinical implementation—most notably, a lack of temporal precision. By applying broad-spectrum, static interventions, previous trials have failed to distinguish between the transient “good” senescence (immunostimulatory) and the persistent “bad” senescence (immunosuppressive).

These insights motivate a Time-Aware Therapeutic Framework (Fig. 4). In the early post-therapy interval, actively exploit the induced immunogenicity [e.g., rational placement of senescence cell–based vaccination or DC priming with ICI] [121]. In the intermediate phase, selectively attenuate suppressive SASP modules [e.g., JAK/STAT-directed senomorphic approaches] [19, 128]. Before inhibitory remodeling becomes entrenched, debulk the residual senescent burden with senolysis and deploy layered blockade of suppressive circuits [PD-1/PD-L1, PD-L2–RGMb, CD47–SIRPα, CD73–A2A] [20, 21, 133, 154]. To personalize such strategies, longitudinal multiparametric profiling is needed across the treatment timeline—tracking senescence markers, SASP/senEVs, checkpoint status, myeloid burden, and liquid biopsy signals such as cfDNA-derived DDR signatures and circulating senEV cargo [155–158].

Importantly, the benefit–risk profile of senolysis is context-dependent, varying by tumor type, treatment history, and comorbidities. For example, the BCL-2 inhibitor navitoclax clears senescent cells but has caused thrombocytopenia and neutropenia in patients with advanced small-cell lung cancer [135, 159]. Dasatinib plus quercetin can enhance senescent-cell clearance but has been associated with hematologic abnormalities, QT prolongation, and impaired tissue regeneration [160]. These observations argue for dosing, scheduling, and delivery strategies that preserve benefit while minimizing toxicity [25], together with timing rules that protect the transient immunogenic window created by TISCCs (enhanced neoantigen presentation and innate activation).

Ultimately, TISCCs should be viewed not as indiscriminate debris to be removed, but as programmable immune nodes within combination immunotherapy. Priorities for the field are to: (i) integrate systematic post-treatment reassessment of checkpoints, SASP, senEVs, and MDSC burden into routine clinical decisions; (ii) establish the causal contribution of the TISCC–NETosis axis in preclinical and early clinical studies; (iii) optimize the timing, dose, and delivery of senolysis to ensure safety while maintaining early immunogenic gains; and (iv) develop vaccine strategies that leverage TISCC immunogenicity while rationally combining NETosis and SASP modulation with checkpoint blockade. With this shift, the biological changes induced by standard therapy can be reframed from liabilities into embedded opportunities, enabling more durable cancer control.
